# Design and Implementation of Financial Service and Management Platform considering Support Vector Machine Algorithm

**DOI:** 10.1155/2022/7964123

**Published:** 2022-09-09

**Authors:** Lei Tian

**Affiliations:** School of Economics and Management, Chifeng University, Inner Mongolia, Chifeng, China

## Abstract

With the rapid economic development, the financial industry has quietly become the leader of industries, the core and lifeblood of promoting economic development. At the same time, various financial services and management platforms emerge one after another. However, the emergence of financial services and management platforms cannot effectively alleviate the current financial crisis. In the face of increasingly complex financial risks, traditional financial service and management platforms cannot achieve effective information sharing, which leads to continued low service and management efficiency and frequent financial risk problems. Support vector machine is a data classification algorithm based on supervision, which can realize data sharing and improve the efficiency of data processing. The article firstly readjusted the underlying architecture of the financial service and management platform to break through the barriers of data interaction. Then on this basis, the article further combines the support vector machine algorithm and extends it from binary data classification to multivariate classification. Finally, the paper redesigns the financial service and management platform considering support vector machines. After a series of experiments, it can be found that the financial service and management platform based on the support vector machine algorithm can reduce the financial risk by 17.2%, improve the financial service level by 30.2%, and improve the financial comprehensive service level by 45.2%. At the same time, thanks to information sharing and interaction, the financial service and management platform can effectively predict financial risks, and the accuracy of the prediction basically reaches 78.9%. This shows that a financial service and management platform that takes into account the support vector machine algorithm can effectively prevent financial risks and improve the efficiency of financial services and management.

## 1. Introduction

With the rapid development of the Internet, the financial industry has gradually penetrated into every corner of life and brought a great impact on the traditional financial system. In order to cope with the rapidly developing economy and avoid major financial turmoil, many banks and core enterprises have established their own financial service and management platforms. However, for a long time, the quality of financial services has not been improved because the information between financial institutions and enterprises has not been communicated with each other, and many enterprises have suffered greatly. At the same time, due to the blockage of information, the comprehensive efficiency of financial services and management is low, and financial risks occur frequently. The support vector machine algorithm can fully mine the data between financial institutions and enterprises, promote information sharing between financial services and management platforms, and improve the efficiency of financial services and management. At the same time, by integrating financial resources, relevant institutions can use the platform to improve their ability to monitor, assess, and prevent risks in financial institutions. On this basis, financial institutions can further enhance their authority. At the same time, by building a financial service and management platform that takes into account the support vector machine algorithm, it can further accelerate the innovation of financial service products, improve the relevant system of financial management, enhance the ability of the financial industry to resist risks, and promote the stable and long-term development of the financial field.

After a series of experiments, we knew that the financial service and management platform based on support vector machine can greatly improve the level of financial services. Before the support vector machine is not taken into account, financial services cannot synthesize all information, and the core enterprises provide financial services with low efficiency and single products, and the service level is as low as 11.2%. After focusing on support vector machines, the service level of the platform increased by 30.2% year-on-year, and the comprehensive average service level of the platform increased to 45.2%. In the process of financial risk prediction and assessment, the Bayesian algorithm can effectively predict financial risks caused by supervision with an accuracy of 71.2%. The risk prediction method based on neural network can effectively predict the risk of financial investment with an accuracy of 71.9%. In contrast, the financial service and management platform based on support vector machine can better predict various business risks in the financial field, and its prediction accuracy can reach up to 78.9%. This fully shows that the financial service and management platform based on support vector machine can effectively improve the efficiency of financial services and financial management level and can predict financial risks in a timely manner.

## 2. Related Work

With the continuous development of the financial industry, the problems encountered in financial services and management are becoming more and more serious. Many scholars have discussed this. Chang-Ho pointed out that as the global economy continues to slump, companies with low liquidity and low profitability will face a recession, which is expected to increase the risk of bankruptcy. Liquidity and profitability are closely related in the analysis of operating performance. Therefore, he used a standardized correlation matrix to establish a simple financial service platform to analyze the financial factors that affect the liquidity and profitability of financial enterprises [1]. Dhiman pointed out that the financial and banking industry has made great progress in technology, so he pointed out that it is necessary to deeply study the successful template of digital transformation and quantify it. Taking financial enterprises in the digital age as the research object, he focused on the help of digital technology in the financial field to human decision-making and emphatically analyzed the impact of financial management methods on the economic development of enterprises [2]. Piatti-Fünfkirchen and Schneider pointed out that the mobile financial service platform is an innovation of the traditional financial platform and has strategic significance for the cross-border development of the global financial industry. In the research process, they studied the mobile financial service platform as a complex ecosystem and put forward the development direction and related suggestions of the mobile financial service platform [3]. Omigie et al. analyzed the motivation of financial product innovation in order to explore the risk of banking financial services under the background of informationization. Therefore, they established a new financial product service platform with spillover effects, different competitive strategies, and risk constraints. On the basis of analyzing the risk characteristics of financial product innovation, they discussed the risk transmission mechanism of the financial product innovation chain and proposed a new model of internal risk management and external risk supervision of bank financial product innovation [4]. Ali and Isak's research examined the impact of financial management platforms on financial management mechanisms. During the research process, they used explanatory and descriptive research designs, using stratified sampling techniques to select a sample of 145 respondents [5]. Although the above scholars have analyzed the problems of financial services and management from different levels, they have not found the essence of the problem.

Support vector machines are often used in data classification and mining, which can accurately find problems in financial services and management, and many scholars have also conducted research on this. Guo et al. pointed out that smart hospitals are considered a promising technology and platform that can greatly improve healthcare in the future. In order to further strengthen the platform construction, they analyzed two important parameters of the signal pulse through the support vector machine (SVM) algorithm and accurately divided all cellular events into two different subpopulations. Then the microfluidic sensor is combined with the SVM algorithm to reconstruct the sensor network system of the smart hospital [6]. Hao et al. proposed a comprehensive SVM classification algorithm for the imbalance of training data set. First, they performed unsupervised clustering on the imbalanced training set, then segmented the data set with SVMs to precisely control the local features of the data set, and finally solved the data imbalance [7]. Shyamala et al. pointed out that in the gas pipeline system, the safe operation of the gas pressure regulator determines the stability of the fuel gas supply. Therefore, according to the operating conditions of the medium and low pressure gas regulators in the SCADA system, they proposed a new method for the safety precaution of gas regulators based on SVMs. This method takes the gas pressure regulator outlet pressure data as the input variable of the SVM model and takes the fault type and degree as the output variable, which can effectively improve the prevention accuracy and save a lot of manpower and material resources [8]. Sontayasara et al. pointed out that bearing failure is the main cause of motor and generator failure, so they proposed to use SVM algorithm for early detection and classification of bearing failure. By using his proposed method, bearing failures can be detected at an early stage, giving machine operators time to take preventive measures before large-scale failures occur [9]. Zhu pointed out that the precise analysis of the data is essential to improve the reliability of the diagnostic method. Therefore, based on solid-state micropore microfluidic impedance cytometer, he used the SVM algorithm to detect and enumerate cancer cells in red blood cells [10]. The above experts and scholars have fully exploited the advantages of SVMs, but they have not extended the algorithm to the financial field, and their research on the SVM algorithm is not very thorough.

## 3. Support Vector Machine Algorithms and Financial Services and Management

### 3.1. Financial Services and Management

Finance is an economic activity in a market environment, and it represents the entire process of monetary and capital financing. With the rapid economic development, the financial industry has gradually become a barometer of economic development. Therefore, vigorously developing financial services and management is an important measure to promote economic development [11]. Under the influence of the global economic crisis, many industries around the world have not completely emerged from the shadow of the financial crisis. Among them, financial enterprises are particularly affected. After experiencing many economic crises, people gradually realized the importance of strengthening financial management and innovating financial services. Therefore, the majority of financial enterprises and institutions slowly explored and established their own financial services and management systems. In the traditional financial industry, financial services and financial management are actually two completely different concepts. Therefore, the article will explore its existing problems from two levels.

“Financial services” is a general term for financial institutions to use financial instruments to provide information or other services required for investment. In the process of financial services, financial institutions usually provide necessary information for participants and customers, and then customers carry out a series of financial activities based on the relevant information. However, in the process of information exchange, there are obvious information barriers between financial institutions and customers and participants. On the one hand, most of the information is in the hands of financial institutions and financial core enterprises, and customers cannot directly obtain first-hand information and data [12]. On the other hand, the data and related information of customers and event participants using information to engage in financial activities also remain at the customer level and cannot be finally aggregated into financial institutions. Based on this, the information between financial institutions and customers is completely separated, which greatly increases the risk of financial activities, and also brings bad experience to customers. In general, the backward concept of financial services and the singleness of financial service products are the disadvantages of the old financial service model. It is precisely because of the above disadvantages of traditional financial services that it is of great significance for the majority of financial institutions and core enterprises to continuously enhance the awareness of financial services and improve the level of financial services.

Financial management refers to the whole process in which financial institutions and enterprises supervise and coordinate currency circulation and credit activities in accordance with certain financial activity laws. Under the current economic environment, the majority of financial institutions focus their financial management on credit management. The emphasis on credit management is determined by both historical factors and reality. On the one hand, financial crises in history are often caused by credit crises, so strengthening credit management can prevent problems before they occur. On the other hand, in the current economic environment, in the face of business expansion and performance, a large number of illegal loans have appeared in the majority of financial institutions. But only focusing on one area of management can lead to losing all the game [13]. At the same time, the existing financial management methods cannot adapt to the financial model in the new era. Therefore, the continuous innovation of financial management methods and means is an important measure to achieve the sustainable development of the financial industry.

**Figure 1:**
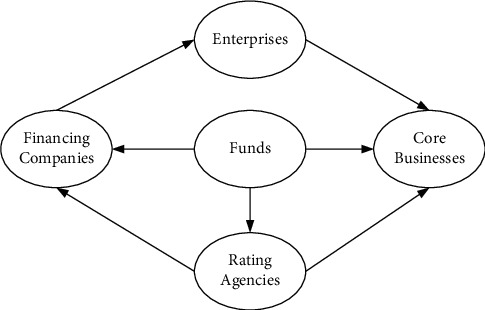
Financial management and service system under the traditional model.

At the level of financial services and financial management, many financial institutions have not fully realized the importance of information exchange and the necessity of establishing a sound management system. At the same time, data and information about the financial sector are basically only circulated among a few core companies. Under such circumstances, the financial services and management systems established by financial institutions and enterprises have inherent drawbacks. Among them, the financial management and service system under the traditional model is shown in Figure 1.

In the traditional financial management and service system, financial institutions and enterprises often focus on the circulation of funds, so funds are often placed in the center of the financial service and management system [14]. In this case, other participants in financial activities often form separate contacts, which greatly reduces the efficiency of service and management and increases the risk of financial activities. In addition, there is a lack of obvious supervision links in the traditional financial management and service system, which has laid hidden dangers for the financial activities of enterprises. Moreover, by observing the architecture, it can be found that the traditional financial model has significant instability, so it is imminent to optimize and upgrade the system.

### 3.2. Financial Services and Management Platforms Focusing on SVM

In the above, the feasibility of combining SVMs with financial services and management platforms is analyzed from the theoretical level. And the utility of the financial service and management platform focusing on SVMs is explored from multiple perspectives. However, there are differences between theory and reality, and the financial cycle and its fluctuations cannot be measured theoretically, so empirical research will be a strong guarantee to test the utility of the platform. At the same time, in order to ensure the accuracy of the data, some data were obtained from the Wande database and the Tai'an database, and the statistical results of the obtained sample data sets are shown in Table 1.

Table 1 shows that the sample data set mainly includes factors such as financial cycle, financial volatility, and economic growth level. Among them, the smaller the financial volatility, the faster the economic growth rate. In order to obtain general laws and methods from general data, the above data sets are added to the training of SVMs. At the same time, in order to further test the correctness of the data classification results of SVMs, this paper uses three different structures of matrices to evaluate the results. The specific empirical results are shown in Table 2.

Table 2 shows that by testing most of the factors, the classification results of the SVM are basically accurate, which lays the foundation for the further experiments of the article. Among them, the financial cycle coefficient remained positive during the three tests and passed the 1% significance level test. This shows that changes in the financial cycle are consistent with the direction of changes in economic growth. In addition, the coefficient of financial volatility is significantly negative in multiple tests, which indicates that there is a negative correlation between it and economic growth.

After the above inspection and evaluation, it is reasonable to believe that the classification and decision-making based on support vectors are accurate. Therefore, people will further examine its role in financial services and management platforms. LR test is an indicator that can best reflect the authenticity of the data, which can reflect the sensitivity and specificity of the model at the same time, so people will use this test method to test the data on the above platforms. Among them, the LR test results of the financial platform based on SVM are shown in Table 3.

Table 3 shows that in the face of different financial risks, the test results of financial platforms based on SVMs are not the same. Among them, technical risks and operational risks are unavoidable during the operation of the platform. Therefore, when dealing with these two types of risks, the LR test value of the platform is relatively high, and the maximum lag value is 11.654. In addition, in the face of liquidity risk and credit risk, the platform's LR test value is low, with a minimum of 5.214. This shows that in dealing with financial risks, the financial platform of the SVM can basically pass the test, and the model selection meets the requirements.

### 3.3. Support Vector Machines

**Figure 2:**
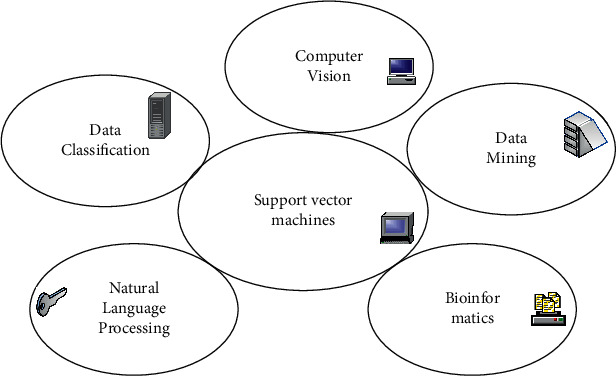
Basic application areas of SVMs.

SVM is often used for data classification and mining, so it can fully mine all the information in the economic environment and classify it to improve the efficiency of information utilization [15]. In the financial environment, information and data are necessary weapons for financial activities, and the emergence of SVMs has provided convenience for the majority of financial institutions. In addition, SVMs not only have excellent characteristics in classification problems, but also have good generalization ability in similar problems. Among them, the basic application areas of SVMs are shown in Figure 2.

**Figure 3:**
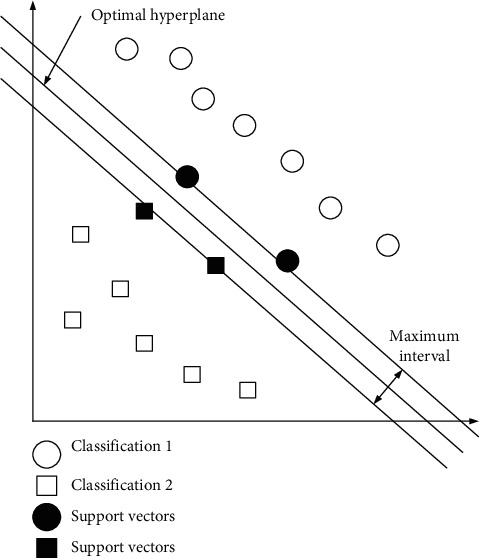
Schematic figure of the integration of SVMs with financial services and management.

With the optimization and upgrading of the SVM algorithm, it has achieved remarkable results in machine vision and natural language processing. In the information age, all production and business activities are inseparable from the support of data, and this is particularly prominent in the financial industry. In particular, financial services and management are financial activities built on data, so the combination of SVMs and financial services and management has a certain information base [16]. In this environment, the SVM can divide the financial market into different categories, then select different financial service and management cases as training data sets, and finally obtain the optimal decision plane and decision function. The schematic figure of the combination of SVM and financial services and management is shown in Figure 3.

In the process of combining technology and reality, SVM has shown excellent scalability, which has laid a solid foundation for the combination of SVM and financial services and management [17]. Among them, from the perspective of geometric structure, the combination of SVM and financial services and management shows strong stability, so this is conducive to fully exploiting and minimizing risks in financial activities. From the perspective of data distribution, the combination of SVMs and financial services and management takes into account both boundary conditions and internal conditions of the sample, so the combination of the two shows strong inclusiveness [18]. It is precisely because the combination of the two realizes the true meaning of learning from each other's strengths and complementing one's weaknesses, so it is particularly important to study them. In the above, the feasibility of combining SVMs with financial services and management is analyzed from the aspects of domain attributes and realistic conditions, and then it will be formally incorporated into the design of financial services and management platforms.

Assuming that there is a linearly separable data set T in the financial activity space, which represents the sample set in the training process, it can be mathematically expressed as

(1) $$\begin{array}{c} \begin{array}{c} T=\left\{\left(a_{1},b_{1}\right),\left(a_{2},b_{2}\right),\left(a_{m},b_{m}\right)\right\},\\ \gamma_{m}=b\left(\frac{\omega }{\omega }∙a_{m}+\frac{b}{w}\right). \end{array} \end{array}$$

Among them, *a* represents the feature vector, *b* represents the pointer, *γ*_*m*_ represents the straight-line distance between the points in the data set, and the positive or negative of *ω* represents the direction of the distance. People specify that positive values represent the positive direction and negative values represent the opposite direction. In this process, information about financial services and management is put into the data set.

Next, people define a hyperplane *s*; then, the minimum value of the distance between the sample point and the plane is calculated as

(2) $$\begin{array}{c} \varepsilon =\min_{i=1,2,3∙∙,M}\gamma_{s}\text{.} \end{array}$$

In the above formula, the s plane is called the hyperplane, so the distance *ε* actually represents the distance from the support vector to the hyperplane. According to the above formulas, the hyperplane can be visualized as a financial market, then the original hyperplane distance problem can be expressed as the following capital circulation optimization problem.

(3) $$\begin{array}{c} y_{i}\left(\frac{\omega }{\omega }∙a_{m}+\frac{b}{w}\right)\geq \gamma \text{.} \end{array}$$

In the above formula, what needs to be explored is the distance between the points in the data set and the plane, that is, the correlation between the financial activity information and the financial market. At the same time, it is expected to use this distance to maximize the segmentation of the data set, so as to find the most valuable information for financial activities. Therefore, for the simplicity of expression, (3) is calculated as follows:

(4) $$\begin{array}{c} y_{i}\left(\frac{\omega }{\omega \gamma }∙a_{m}+\frac{b}{w\gamma }\right)\geq 1\text{,} \end{array}$$

(5) $$\begin{array}{c} \begin{array}{c} \mu =\frac{\omega }{\omega \gamma },\\ b=\frac{b}{w\gamma },\\ y_{i}\left(\mu ∙a_{m}+b\right)\geq 1,\left(m\right.=1,2,...,M. \end{array} \end{array}$$

In the above formula, first, on the basis of (3), both sides are divided by *γ*, and then (4) is obtained. Then the scalars *ω* and *γ* are replaced by the vectors *μ* and b through a series of iterative calculations, thereby obtaining (7). After the above operations, the massive information is divided into different categories, and the same data are optimized.

The premise of the above optimization problem is a binary classification object, but in the process of actual financial activities, multiclassification problems are often encountered. Quadratic programming can transform multiclassification problems into binary classification, from which people can get

(6) $$\begin{array}{c} L\left(\omega ,b,\alpha \right)=\frac{1}{2}\omega^{2}-\sum_{i=1}^{M}\alpha_{i}\left(y_{i}\left(\mu ∙a_{m}+b\right)-1\right)\text{,} \end{array}$$

(7) $$\begin{array}{c} \theta \left(\omega \right)=\max_{\alpha_{i}\geq 0}L\left(\omega ,b,\alpha \right)\text{.} \end{array}$$

Among them, *L* represents the Lagrangian function, and *α* represents the Lagrangian multiplier. Observing (6), it can be found that there are three variables in the newly constructed objective function, but there are only two pairs of existing constraints, so the optimal solution of the function cannot be solved only by the above formula.

Using Lagrange's duality, (6) can be reformulated as

(8) $$\begin{array}{c} \begin{array}{c} \max_{\alpha_{i}\geq 0}\min_{\omega ,b}L\left(\omega ,b,\alpha \right)=c^{*},\\ \omega =\sum_{i=1}^{M}\alpha_{i}y_{i},\\ b=\overset{M}{\underset{i=1}{\sum }}\alpha_{i}y_{i}=0. \end{array} \end{array}$$

In the above formula, the original optimal solution problem can be transformed into the problem of solving the maximum value. Similarly, for similar maximum segmentation problems, we can transform it into finding the maximum value.

By solving the maximum value, the optimal solution for the current situation can be obtained:

(9) $$\begin{array}{c} \begin{array}{c} \alpha^{*}={\left(a_{1}^{*},a_{2}^{*},...,a_{m}^{*}\right)}^{T},\\ 0\leq a^{*}\leq d,\\ a\left(x\right)=sign\left(\omega^{*}∙x+b\right). \end{array} \end{array}$$

In the above formula, *α*^*∗*^ represents the optimal solution, and *α* represents the optimal classification decision function. Through a series of calculations, people can finally use the data in financial activities to carry out financial services and get the most favorable decision-making plan.

In the above, the data set defined by people is linearly separable, but in practice there are very few completely linearly separable data in financial activities. To solve this problem, the concept of soft spacing is introduced.

(10) $$\begin{array}{c} \begin{array}{c} \min_{\omega ,b,\delta }\frac{1}{2}\omega^{2}+d\overset{m}{\underset{i=1}{\sum }}\delta_{i},\\ y\left(\omega ∙a_{m}+b\right)\geq 1-\delta_{i},\\ \delta_{i}=\max\;\left(0,1-y\left(\omega ∙a_{m}+b\right)\right). \end{array} \end{array}$$

Among them, *δ* represents a random variable, which allows some points in the data set to not meet the basic constraints, and *d* represents the penalty function: the larger the value, the greater the penalty for the classification result. Then people perform a separation operation on the hyperplane:

(11) $$\begin{array}{c} \omega^{*}\cdot x+b=0\text{.} \end{array}$$

Finally, for the above nonlinear problem, one can refer to the linear solution problem and solve it using the duality of Lagrangian. Then the optimal solution is obtained and the decision function is finally positioned to obtain the best solution for financial services and management. Through the above operations, it is also found that for the financial service and management data set *T*, none of the training data samples in the final solution model appear. This fully demonstrates the excellent characteristics of SVMs; that is, the final result is only related to support vectors, and it is also greatly adapted to financial services and management activities.

### 3.4. Design of Financial Service and Management Platform Focusing on Support Vector Machines

**Figure 4:**
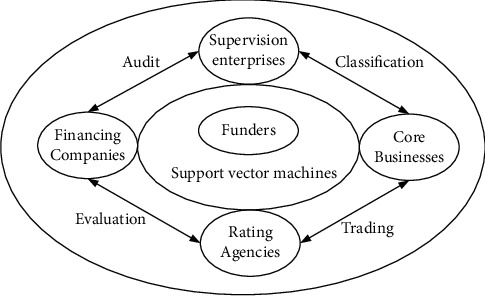
Financial service and management platform under the SVM algorithm.

Reviewing the traditional financial service and management system, it is found that it has not formed a complete information channel and lacks effective supervision and management in the process of financial activities. Therefore, in order to maximize the effectiveness of the combination of SVMs and financial services and management, the traditional financial services and management systems have been optimized and upgraded. Then a financial service and management platform considering SVMs is designed. The financial service and management platform under the SVM algorithm is shown in Figure 4.

On the basis of the traditional financial service and management system, we use the SVM algorithm to reoptimize and adjust it. The following is the specific design and implementation process. First, in the original financial service and management system, people retained the basic core financial enterprises and financial structures in the financial service and management platform under the SVM algorithm [19]. In the financial market environment, financial enterprises and financial structures are the main body of the market environment. Therefore, no changes were made to the two during the design process. However, in view of the problem of noncommunication of information between various subjects, SVMs are used to bind the basic subjects in the financial market to data to achieve basic information communication. At the same time, in order to adapt it to the new financial service and management platform, it also provides data classification and tracking rights for these two subjects. Secondly, in the traditional financial service and management system, the financial supervision link is not prominent. Therefore, people have joined the supervision department in the financial service and management platform under the SVM algorithm. And in the design, people directly make it independent of the entire financial services and management links to achieve the autonomy of supervision [20]. Finally, in order to realize the exchange of information and promote the sharing of information among various subjects, a bidirectionally visible information module was added at the beginning of the platform design, which really improved the utilization rate of information from the bottom of the design. At the same time, in order to meet the financial market environment, the central position of funds in the financial service management system has been cancelled. Instead, it puts customers and investors at the center of the financial service and management platform to optimize the original system to the greatest extent and realize innovation in financial services and management. Moreover, the redesigned financial services and management platform achieves structural stability, which will aid its continued growth.

At the same time, in the implementation process of the financial service and management platform, the platform makes full use of SVMs to train and mine financial data. Then, according to the results, it can accurately profile financial institutions and customers, so as to help small- and medium-sized enterprises and small and micro enterprises to carry out financial activities [21]. In the process of profiling the enterprise, the relevant information of the enterprise will be circulated on the platform, so the enterprise applying for financial services can spend the least time to maximize the benefits. At the same time, financial institutions can also publish financial products and services on the financial service and management platform, and enterprises that meet relevant conditions can apply directly on the platform to improve the efficiency and experience of financial services. For financial institutions, the application information of enterprises will be circulated directly on the platform, which avoids the leakage of information from multiple parties, and at the same time, it can further improve the efficiency of financial management. In this process, the financial service and management platform is customer-oriented, based on financial activity data, and uses SVMs and modern technology as means to build a multi-integrated, information-interoperable financial service and management model.

## 4. Deconstruction of Financial Services and Management Platform Results considering SVM

**Figure 5:**
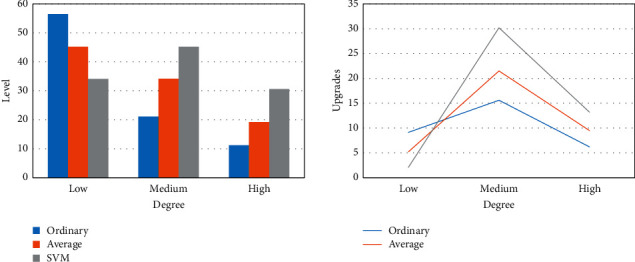
Financial services levels considering SVM.

In the above, the financial service and management platform based on SVM has been empirically researched, but the theoretical test and pure data test cannot explain its effect in practical application. Therefore, the following article will explore the efficacy of the financial service and management platform one by one from the perspectives of the financial platform's service level, management accuracy, and risk assessment level. Among them, the financial service level taking into account the SVM is shown in Figure 5.

Figure 5 shows that the financial service and management platform based on SVM can greatly improve the level of financial services. Before the SVM is not taken into account, financial services cannot synthesize all information, the core enterprises provide financial services with low efficiency and single products, and the service level is as low as 11.2%. After focusing on SVMs, the service level of the platform increased by 30.2% year-on-year, and the comprehensive average service level of the platform increased to 45.2%.

**Figure 6:**
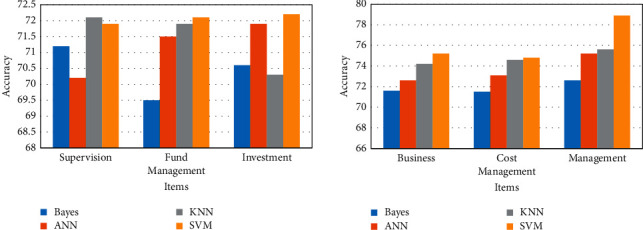
Prediction results of financial risk by different algorithms.

The financial service level is only one part of the financial service and management platform, and it cannot describe the whole figure of the platform. Therefore, from the perspective of financial risk prediction and evaluation, the utility of financial service and management platform focusing on SVM in financial risk prediction is analyzed. The prediction results of different models on financial risk are shown in Figure 6.

In the financial field, the emergence of financial risks is often not without a trace. Figure 6 shows that in the process of financial risk prediction and assessment, the Bayesian algorithm can effectively predict financial risks caused by supervision, with an accuracy of 71.2%. The risk prediction method based on neural network can effectively predict the risk of financial investment with an accuracy of 71.9%. In contrast, the financial service and management platform based on SVM can better predict various business risks in the financial field, and its prediction accuracy can reach up to 78.9%.

**Figure 7:**
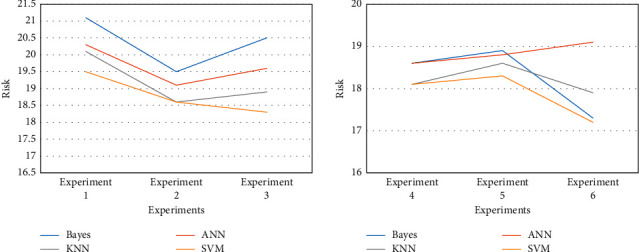
Risk assessment in financial management.

In the process of financial management and services, financial risks are everywhere. In the above, we have conducted experiments on the risk prediction ability of the platform. However, merely predicting risks cannot provide substantial help for subsequent financial investment behaviors. Therefore, it is necessary to evaluate the financial risks predicted by different platforms and conduct supervision and response according to the results of the evaluation, so as to minimize the risks. Among them, the risk assessment results of different platforms are shown in Figure 7.

Figure 7 shows that platforms based on different models have different risk factors. Among them, the platform risk coefficient based on the K series model is generally low, basically 18.3, and the platform risk based on the Bayesian model is relatively high, reaching 20.5%. In contrast, platforms based on SVMs have a lower risk factor of 17.2%.

**Figure 8:**
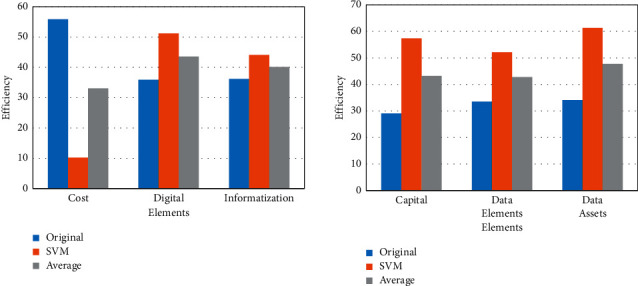
Comprehensive efficiency of financial platform based on SVM.

The financial service and management platform is relatively comprehensive, so it is necessary to analyze its efficiency from a comprehensive perspective. Among them, the comprehensive efficiency of the support vector machine-based financial service and management platform is shown in Figure 8.

The comprehensive efficiency of the financial platform is the efficiency of the financial sector serving the real economy. Figure 8 shows that the support vector machine-based financial service and management platform can amplify the role of data capital and promote the flow of funds across the industry. Among them, the financial service and management platform based on SVM can reduce the cost of trial and error, and its cost consumption is basically kept below 10.2%. In addition, the financial platform comprehensively utilizes multiparty information to realize the informatization and digitization of the platform, and its comprehensive proportion is 44.1% and 51.2%, respectively. This fully shows that the financial service and management platform based on SVM can comprehensively improve the efficiency of financial services and the level of financial management.

## 5. Conclusion

Financial risk is a major problem inevitably encountered in the development of the financial industry, and the breakthrough is to build a new financial service and management platform for information communication and business interaction. The article starts with the inherent problems of financial services and management and briefly analyzes the shortcomings and deficiencies of traditional financial services and management platforms. Then, the article optimizes and upgrades the method based on the SVM and integrates the SVM algorithm into the design of the financial service and management platform. Finally, the article focuses on the related methods and countermeasures of using SVMs to improve financial services and management. Experiments show that a financial service and management platform that considers support vectors can innovate financial services and management and promote the sustainable development of the financial industry. However, due to time reasons, the financial service and management platform designed in this article still has some deficiencies in system security. In the future, the article will improve this aspect in the process of upgrading and optimizing the platform.

## Figures and Tables

**Table 1:** Sample data set statistics.

Variables	Average value	Variance	Minimum value	Maximum value

Cycle	0.768	0.611	−0.098	2.115
Vol	0.091	0.083	0.006	0.732
InGDP	3.642	0.381	1.002	4.216
Capital	0.492	0.151	0.209	1.395

**Table 2:** Empirical results under multiple factor assessment.

Variables	Adjacency matrix	Diagonal matrix	Inverse matrix
Models	SVM	SVM	SVM
Cycle	0.231^*∗∗∗*^	0.038^*∗∗∗*^	0.019^*∗∗∗*^
Vol	−0.035	−0.029^*∗∗*^	−0.034^*∗∗∗*^
InDA	0.0139	0.0115	0.0451^*∗*^
Capital	0.031^*∗*^	0.019	0.214

*Note.* ^*∗*^, ^*∗∗*^, and ^*∗∗∗*^represent data at 10%, 5%, and 1% confidence levels, respectively.

**Table 3:** Support vector machine-based financial platform LR test.

	Credit risk	Liquidity risk	Operational risk	Technical risk
Hysteresis	11.058	5.214	9.021	10.654
Error	12.021	8.399	11.654	9.687
Regulation	16.215	10.005	18.001	2.134

## Data Availability

The data used to support the findings of this study can be obtained from the author upon request.
